# Effect of Vitamin C/Hydrocortisone Immobilization within Curdlan-Based Wound Dressings on In Vitro Cellular Response in Context of the Management of Chronic and Burn Wounds

**DOI:** 10.3390/ijms222111474

**Published:** 2021-10-25

**Authors:** Michal Wojcik, Paulina Kazimierczak, Vladyslav Vivcharenko, Malgorzata Koziol, Agata Przekora

**Affiliations:** 1 Independent Unit of Tissue Engineering and Regenerative Medicine, Medical University of Lublin, Chodzki 1 Street, 20-093 Lublin, Poland; michal.wojcik@umlub.pl (M.W.); paulina.kazimierczak@umlub.pl (P.K.); vlad.vivcharenko@gmail.com (V.V.); 2 Department of Medical Microbiology, Medical University of Lublin, Chodzki 1 Street, 20-093 Lublin, Poland; malgorzata.koziol@umlub.pl

**Keywords:** β-1,3-glucan, cell migration, biomaterials, skin regeneration, matrix metalloproteinases

## Abstract

Bioactive dressings are usually produced using natural or synthetic polymers. Recently, special attention has been paid to β-glucans that act as immunomodulators and have pro-healing properties. The aim of this research was to use β-1,3-glucan (curdlan) as a base for the production of bioactive dressing materials (curdlan/agarose and curdlan/chitosan) that were additionally enriched with vitamin C and/or hydrocortisone to improve healing of chronic and burn wounds. The secondary goal of the study was to compressively evaluate biological properties of the biomaterials. In this work, it was shown that vitamin C/hydrocortisone-enriched biomaterials exhibited faster vitamin C release profile than hydrocortisone. Consecutive release of the drugs is a desired phenomenon since it protects wounds against accumulation of high and toxic concentrations of the bioactive molecules. Moreover, biomaterials showed gradual release of low doses of the hydrocortisone, which is beneficial during management of burn wounds with hypergranulation tissue. Among all tested variants of biomaterials, dressing materials enriched with hydrocortisone and a mixture of vitamin C/hydrocortisone showed the best therapeutic potential since they had the ability to significantly reduce MMP-2 synthesis by macrophages and increase TGF-β1 release by skin cells. Moreover, materials containing hydrocortisone and its blend with vitamin C stimulated type I collagen deposition by fibroblasts and positively affected their migration and proliferation. Results of the experiments clearly showed that the developed biomaterials enriched with bioactive agents may be promising dressings for the management of non-healing chronic and burn wounds.

## 1. Introduction

Occurrence of some factors (e.g., infection, diabetes, arthritis) may disturb or delay the normal healing process, leading to chronic wounds [1]. The chronic wounds are characterized by prolonged inflammation and healing, elevated levels of matrix metalloproteinases (MMPs) and reactive oxygen species (ROS), reduced level of pro-healing growth factors (e.g., transforming growth factor-beta 1—TGF-β1, vascular endothelial growth factor–VEGF), senescent cell populations, lack of microvasculature, and susceptibility to infections [2,3]. Proper treatment and care of non-healing wounds may overcome their molecular and cellular deficiencies. Common technologies applied for the treatment of chronic wounds include negative pressure wound therapy, hyperbaric oxygen therapy, electrical stimulation, and various cellular/acellular therapies [3]. Nevertheless, every wound care technology requires also application of appropriate wound dressing, which plays a crucial role in the regeneration process. Wound dressings should protect a wound bed against foreign bodies and microbes, as well as provide an appropriate moist environment and remove excessive wound exudate. Additionally, wound dressing should exhibit low adherence to the wound bed protecting against destruction of a newly formed tissue during dressing exchange. Mentioned properties of the dressing material support appropriate wound healing process [4,5].

Wound dressings are usually produced using natural or synthetic polymers. Recently special attention has been paid to β-glucans that act as immunomodulators and have pro-healing properties. Curdlan is a linear bacterial β-1,3-glucan produced by *Alcaligenes faecalis*. It is non-toxic polymer with proven anti-infective and pro-healing activities [6]. There are many dressings available on the market, which occur in various forms and have different applications; e.g., films, hydrogels, hydrocolloids, hydrofibers, foams [2]. Nevertheless, nowadays, there has been a growing interest in the development of bioactive wound dressings which exhibit active participation in the wound healing process by sustained release of the bioactive compounds immobilized within the dressing structure [7]. The bioactive wound dressings may contain drugs/antibiotics (e.g., ibuprofen, lidocaine, ciprofloxacin, tetracycline) [8,9,10,11], vitamins (e.g., vitamin B12, vitamin C) [12,13], ions (e.g., zinc, silver) [14,15], growth factors/cytokines (e.g., fibroblast growth factor–FGF, epidermal growth factor–EGF, VEGF, platelet-rich plasma) [4,16], plant-derived compounds (such as curcumin and essential oils) [17,18,19] or cells (e.g., mesenchymal stem cells) [4]. In contrast to passive dressings, bioactive dressings provide a favorable environment improving and accelerating wound healing [7].

In our previous research [20], two variants of hybrid curdlan-based dressings were developed: curdlan/agarose (marked as Mat1 throughout this manuscript) and curdlan/chitosan (marked as Mat2 throughout this manuscript) which were characterized by hydrocolloids properties, foam-like structure with superabsorbent ability, as well as non-toxicity against human skin fibroblasts. Moreover, surfaces of developed biomaterials were unfavorable to cell adhesion which is a crucial feature during dressing exchange, enabling painless material removal after completed healing. It is noteworthy that both Mat1 and Mat2 are newly developed biomaterials with innovation characteristics (Polish Patent no. 236367 and patent application no. P.430455, respectively). The main objective of this study was to produce bioactive curdlan-based wound dressings enriched with vitamin C, hydrocortisone, and a mixture of both mentioned bioactive substances to accelerate the healing of chronic and burn wounds. Vitamin C (ascorbic acid) is a well-known water-soluble antioxidant that plays a crucial role in skin regeneration by promotion of collagen synthesis, angiogenesis, and antioxidant protection of the skin [21]. In turn, hydrocortisone is a corticosteroid that has potential to suppress immune and inflammatory responses [22]. Hydrocortisone is used for the treatment of chronic wounds in burn patients when hypergranulation tissue is formed [23]. Thus, it was hypothesized that biomaterials enriched with vitamin C and/or hydrocortisone may be potential candidates to be used as a dressing materials for the management of chronic wounds (e.g., diabetic ulcers, venous and arterial ulcers) and non-healing burn injuries, which are characterized by prolonged inflammation, excessive levels of proteases, hypoxia, lack of microvasculature, and a high level of ROS [3].

Within the studies, vitamin C and hydrocortisone release profile from developed curdlan-based biomaterials was determined. Moreover, the impact of released drugs on in-vitro cellular response was evaluated: (1) cytotoxicity, (2) fibroblast proliferation, (3) fibroblast and keratinocyte migration, (4) deposition of type I collagen (Col I) by fibroblasts, and (5) synthesis of MMP-2 and TGF-β1 by skin cells and macrophages. All cell culture experiments were conducted in a two-compartment environment using cell culture inserts since surfaces of produced biomaterials prevented cell adhesion. Performed studies allowed to answer the question whether modification of previously developed curdlan-based biomaterials with bioactive compounds improves their biological properties and makes them optimal dressing materials for acceleration of the healing process during management of chronic and burn wounds.

## 2. Results and Discussion

### 2.1. Vitamin C and Hydrocortisone Release Profile

**Figure 1:**
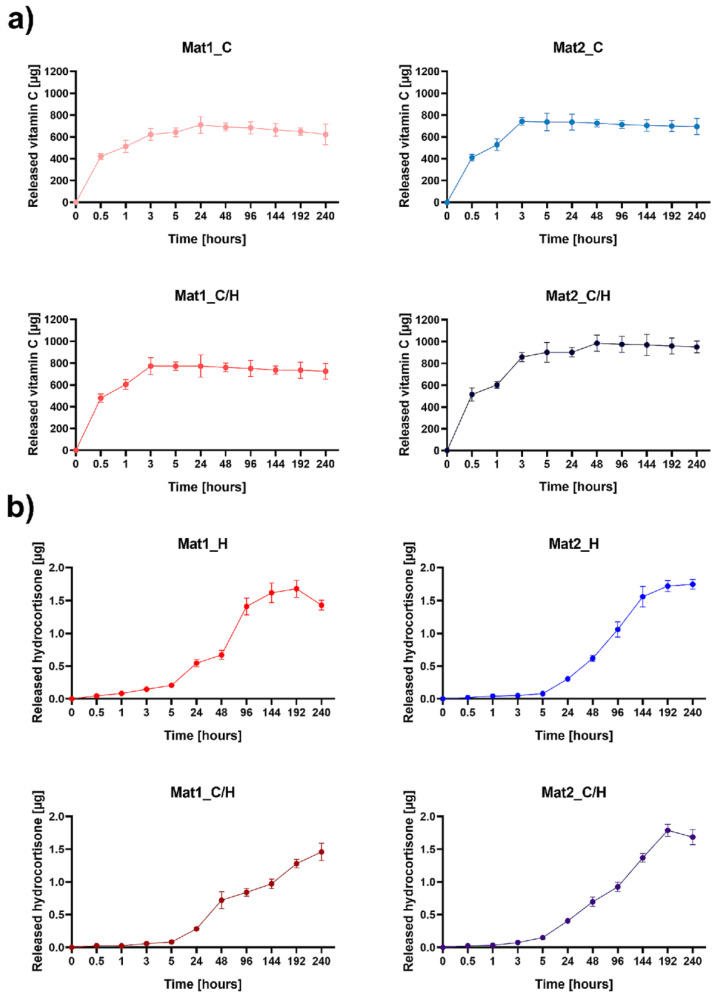
The cumulative release profile of (**a**) vitamin C and (**b**) hydrocortisone from curdlan-based biomaterials (Mat1: curdlan/agarose biomaterial, Mat2: curdlan/chitosan biomaterial, C–vitamin C, H–hydrocortisone, C/H–mixture of vitamin C and hydrocortisone).

The vitamin C and hydrocortisone release from the curdlan-based biomaterials was evaluated over 10 days using drug release USP4 Sotax apparatus with a closed-loop system (Figure 1). It was observed that the developed biomaterials exhibited higher and faster vitamin C profile release than hydrocortisone. As shown in Figure 1a, the Mat1_C/H and Mat2_C exhibited burst release of the vitamin C within the first 3 h, whereas Mat1_C within 24 h and Mat2_C/H released vitamin C until 48 h, then showed a plateaued release of vitamin C. Interestingly, Mat2_C/H released a noticeably greater amount of vitamin C (approx. 985 µg) compared to other biomaterials modified with vitamin C (in the range of 704–772 µg). The burst release of vitamin C from biomaterials is a good property since it may cause rapid scavenging of ROS what has a beneficial impact on the proper healing of chronic and burn wounds, which are characterized by the overproduction of these species [24]. Unlike vitamin C, curdlan-based biomaterials modified with hydrocortisone displayed slow and gradual release of hydrocortisone (Figure 1b). Apart from Mat1_C/H, all hydrocortisone-enriched biomaterials (Mat1_H, Mat2_H and Mat2_C/H) achieved plateau in hydrocortisone release after 192 h (8 days). Importantly, all biomaterials released similar amount of hydrocortisone (approx. 1.75 µg).

The gradual release of low concentrations of hydrocortisone is much desired since it enables topical delivery of therapeutic agent, minimalizing systemic side effects. It is worth noting that low-dosage topical administration of hydrocortisone to the chronic wounds was demonstrated to accelerate healing, reduce pain and exudate release, and primarily to suppress the development of hypergranulation tissue [25]. Jaeger et al. [23] proposed topical application of gauze pad soaked in a hydrocortisone solution onto the burn wounds with developed hypergranulation tissue for maximum 8 days. Due to burst release of the steroid, they changed the dressing every 12 h. Developed curdlan-based dressings showed gradual release of low doses of the hydrocortisone for 6–8 days beginning from the 1st day. Therefore, application of curdlan-based dressings enriched with hydrocortisone will allow for safe treatment of burn wounds with hypergranulation tissue without necessity of frequent dressing exchange. Furthermore, consecutive release of the steroid and vitamin C from the biomaterials containing the mixture of bioactive agents will protect the wound bed against accumulation of high and toxic concentrations of mentioned drugs [26].

### 2.2. Cytotoxicity and Proliferation Assessment

**Figure 2:**
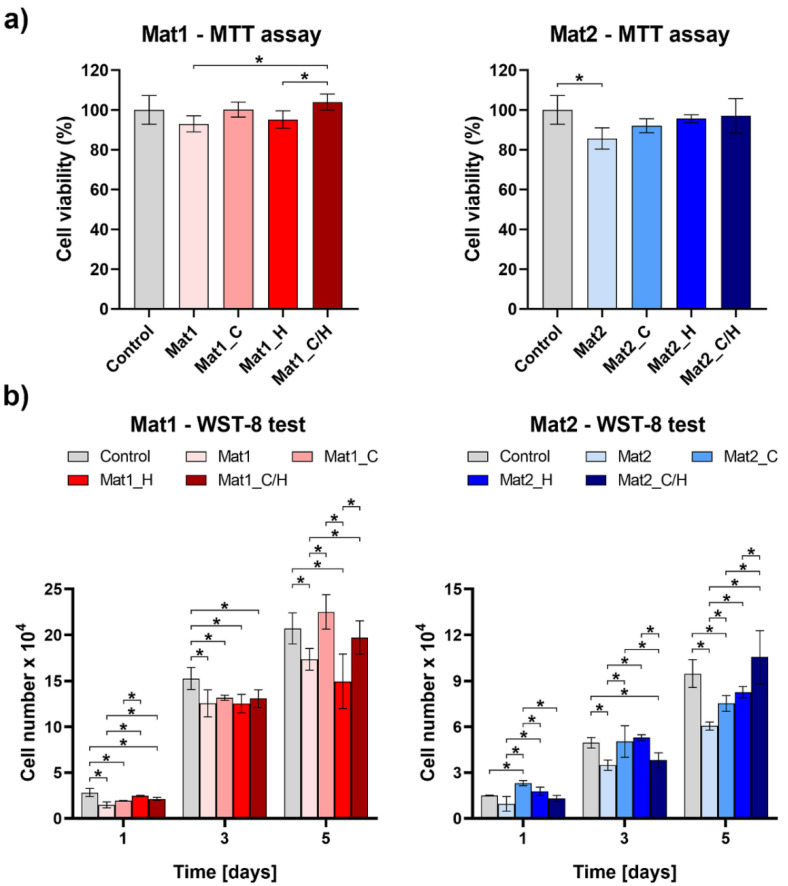
Response of human skin fibroblasts (BJ cell line) to biomaterials: (**a**) cell viability assessment after exposure to 24-h extracts of biomaterials (control–polystyrene extract); (**b**) cell proliferation assessment in a two-compartment environment using cell culture inserts (control–BJ cells cultured in the inserts without any biomaterial); * statistically significant results between indicated groups, *n* = 3, *p* < 0.05, one-way ANOVA followed by Tukey’s test (Mat1: curdlan/agarose biomaterial, Mat2: curdlan/chitosan biomaterial, C–vitamin C, H–hydrocortisone, C/H–mixture of vitamin C and hydrocortisone).

Dressings for wound healing treatment should be biocompatible to avoid irritation and allergic skin response [27]. The cytotoxicity test was conducted in accordance with the procedure described in ISO 10993-5 standard using MTT assay. The test revealed that produced curdlan-based biomaterials were non-toxic to human fibroblasts (BJ cell line) (Figure 2a). Cell viability after exposure to 24-h extracts of biomaterials was above 85% compared to the control cells exposed to polystyrene extract. Moreover, statistically significant increase in cell viability was observed after exposure to extract of Mat1_C/H compared to extracts of Mat1 and Mat1_H. Extract of Mat1_C also increased cell viability compared to Mat1, but no statistically significant differences were reported. It indicates that released vitamin C from Mat1_C and Mat1_C/H had positive effect on fibroblast viability. Although enrichment of Mat2 with vitamin C and hydrocortisone also had a positive effect on cell viability, no statistically significant results were observed.

Impact of produced biomaterials on fibroblast proliferation was assessed by indirect method using cell culture inserts. After 5 days of culture it was observed that vitamin C-enriched variants of Mat1 biomaterial (Mat1_C and Mat1_C/H) significantly improved cell proliferation compared to Mat1 and Mat1_H biomaterials (Figure 2b). Addition of hydrocortisone to the Mat1 biomaterial had a negative effect on cell proliferation. Surprisingly, Mat2 biomaterial reduced cell proliferation compared to the control (cells cultured in the insert without any biomaterial). However, modification of Mat2 with bioactive agents overcame this negative effect on cell proliferation. On the 5th day of the experiment, there were significantly more BJ cells cultured in the presence of Mat2_C, Mat2_H, and Mat2_C/H compared to the unmodified Mat2 sample. Therefore, in the case of Mat2 improved fibroblast proliferation resulted from the presence of both vitamin C and hydrocortisone in the cell microenvironment. Vitamin C is well known factor that enhances proliferative phase of the cells [28], whereas low dosages of hydrocortisone are known to accelerate healing [25]. Hydrocortisone had positive effect on cell proliferation only after its immobilization within Mat2 biomaterial that was produced using acetic acid as a solvent and showed slightly acidic pH of 5.92 (Mat1 was prepared using water and revealed pH of 6.93) [20]. Das Gupta [29] demonstrated that hydrocortisone shows the best stability at acidic aqueous solutions (optimal pH = 3.5). Thus, better biological activity of the hydrocortisone after incorporation within Mat2 structure may be explained by better steroid stability at slightly acidic pH of the biomaterial.

### 2.3. Cell Migration Assessment

**Figure 3:**
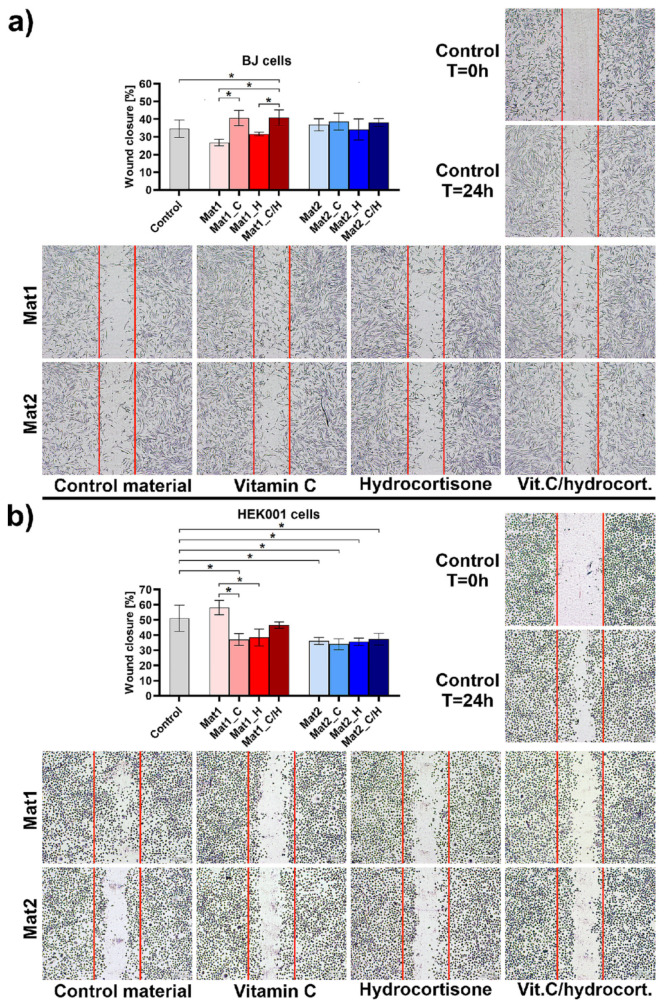
Cell migration after exposure to the extracts of biomaterials: (**a**) migration of human skin fibroblasts (BJ cell line) and (**b**) migration of human epidermal keratinocytes (HEK001 cell line) (control–cells maintained in the presence of polystyrene extract); * statistically significant results between indicated groups, *n* = 3, *p* < 0.05, one-way ANOVA followed by Tukey’s test (Mat1: curdlan/agarose biomaterial, Mat2: curdlan/chitosan biomaterial, C–vitamin C, H–hydrocortisone, C/H–mixture of vitamin C and hydrocortisone, T = 0 h–wound surface just after scratching, T = 24 h–wound surface 24 h after scratching).

Fibroblasts and keratinocytes are crucial cells participating in skin wound healing. During cutaneous wound healing, dermal fibroblasts and keratinocytes migrate into the wound bed and proliferate, closing the wound. Fibroblasts synthesize ECM and support the formation of new blood vessels, creating granulation tissue. In turn, keratinocytes re-create a functional epidermis which protects underlying tissues from trauma [1]. In this study, the impact of curdlan-based biomaterials on fibroblasts (BJ cell line) and keratinocytes (HEK001 cell line) migration was evaluated by wound healing assay using extracts of the materials. Obtained results were expressed as the percentage of the wound closure at 24 h post-scratch compared to the control cells (cultured in polystyrene extract). As shown in Figure 3a, extracts of Mat1_C and Mat1_C/H (wound closure was equal to 40.68 and 40.88%, respectively) significantly stimulated fibroblast migration compared to the control cells (34.62%) and other extracts (biomaterials without vitamin C). Surprisingly, Mat2 biomaterial as well as its modified variants had no effect on fibroblast migration. In the case of keratinocytes, all modified (enriched with bioactive agents) variants of Mat1 and all variants of Mat2 negatively affected keratinocyte migration compared to the control cells (Figure 3b). However, mentioned extracts did not completely inhibit keratinocyte mobility, but slightly slowed down their migration. It should be noted that Sharpe et al. [30] demonstrated that the optimal pH for keratinocyte migration is 8.55. Thus, reduced mobility of keratinocytes after exposure to biomaterials most likely resulted from decreased pH in the cell microenvironment due to the presence of bioactive agents or acetic acid used for the production of the Mat2 dressing.

### 2.4. Type I Collagen Production Assessment

ECM components, such as collagen, fibronectin, vitronectin, proteoglycans, glycosaminoglycans, play a pivotal role in the wound healing. Mentioned biomolecules are responsible for a complex interaction between ECM and cells (monocytes/macrophages, neutrophils, fibroblasts, keratinocytes, and endothelial cells), which provide restoration of tissue integrity [31]. Collagen is the major component of skin ECM, constituting approximately 70% of all biomolecules. Collagen protein not only contributes to skin remodeling but also modulates cellular inflammatory response [32]. Fibroblasts are abundant cells present in the skin that actively produce ECM proteins, including collagen [33].

**Figure 4:**
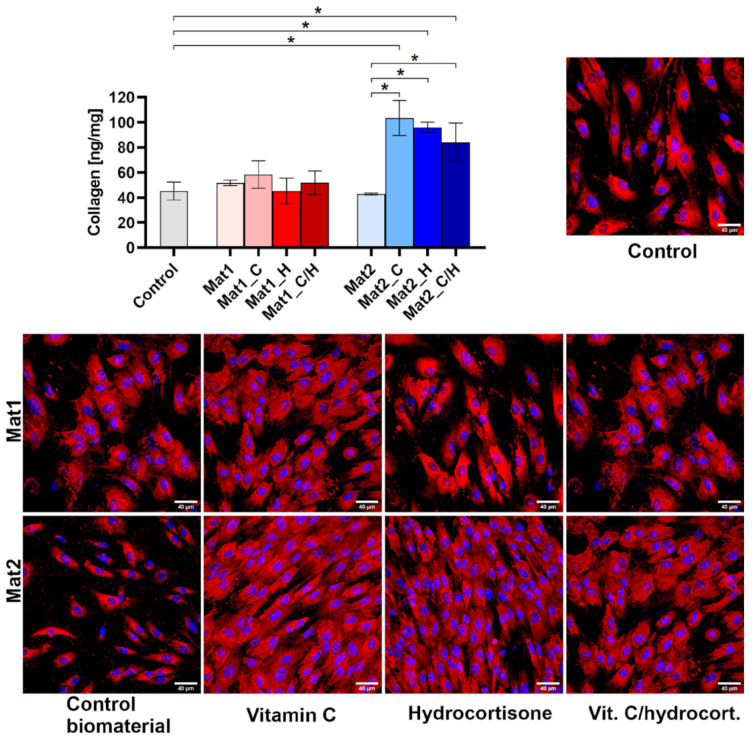
Quantitative (ELISA) and qualitative (immunofluorescence staining) assessment of type I collagen synthesis by human fibroblasts (BJ cell line) after culture in the presence of biomaterials (control–cells culture in the inserts without biomaterial, images: collagen–red fluorescence, nuclei–blue fluorescence, magnification 200×, scale bar = 40 µm); * statistically significant results between indicated groups, *p* < 0.05, one-way ANOVA followed by Tukey’s test (Mat1: curdlan/agarose biomaterial, Mat2: curdlan/chitosan biomaterial, C–vitamin C, H–hydrocortisone, C/H–mixture of vitamin C and hydrocortisone).

In this study, the level of Col I synthesis by human fibroblasts (BJ cell line) was determined after cell culture in the presence of produced biomaterials. As shown in Figure 4, Mat1 and its modified variants containing bioactive agents did not have any impact on collagen synthesis. However, addition of bioactive agents to Mat2 (Mat2_C, Mat2_H, and Mat2_C/H) resulted in a significant increase in Col I synthesis compared to the cells cultured in the presence of Mat2 and control cells. Quantitative results obtained with ELISA were also confirmed by immunofluorescent staining of Col I (Figure 4). It is well known that vitamin C, which is a pivotal cofactor for enzymes during collagen synthesis, participates in the regulation of collagen secretion into the ECM [34]. Interestingly, performed experiment revealed that Mat2_H containing only hydrocortisone also significantly increased Col I synthesis. Prolonged and continuous application of hydrocortisone (systemic, topical, and inhaled) is known to decrease collagen deposition in the skin [35]. Nevertheless, Nuutinen et al. [36] showed that intermittent hydrocortisone treatment may have positive effect on collagen production by the skin cells. Thus, short-term application of developed hydrocortisone-enriched dressings, revealing slow steroid release profile, may have positive clinical outcomes. Lack of stimulation of Col I production by Mat1 enriched with bioactive agents may be explained by poor stability of the hydrocortisone and vitamin C at pH close to the neutral (6.93 for Mat1 biomaterial). Unlike Mat1, Mat2 biomaterial was produced using acetic acid and thus provided better stability of steroid and ascorbic acid [29,37].

### 2.5. MMP-2 and TGF-β1 Production Assessment

Interaction between ECM components and cells within skin is regulated by numerous biochemical mediators, growth factors, and cytokines, such as interferons, interleukins, tumor necrosis factor-alpha (TNF-α), platelet derived growth factor (PDGF), FGF, EGF, VEGF, or TGF-β. Mentioned factors assist in healing process by regulation of inflammatory response, cell proliferation, and differentiation [31]. Moreover, MMPs, such as gelatinases, collagenases, stromelysins, metalloelastase, also play an important role in the wound healing [38]. MMPs are expressed by fibroblasts, keratinocytes, monocytes, macrophages, lymphocytes, and endothelial cells within the skin tissue [39]. The primary role of MMPs during all phases of the wound healing is to remove damaged and temporary components (e.g., fibronectin, fibrin, type III collagen) of ECM, which is called tissue remodeling. Nevertheless, elevated MMP activity is responsible for the development of a chronic wound [39,40]. In this study, the level of MMP-2 (gelatinase A) and TGF-β1 synthesis by human fibroblasts (BJ cell line), human keratinocytes (HEK001 cell line), and human macrophages (derived from THP-1 cell line) cultured in the inserts in the presence of produced biomaterials was determined.

**Figure 5:**
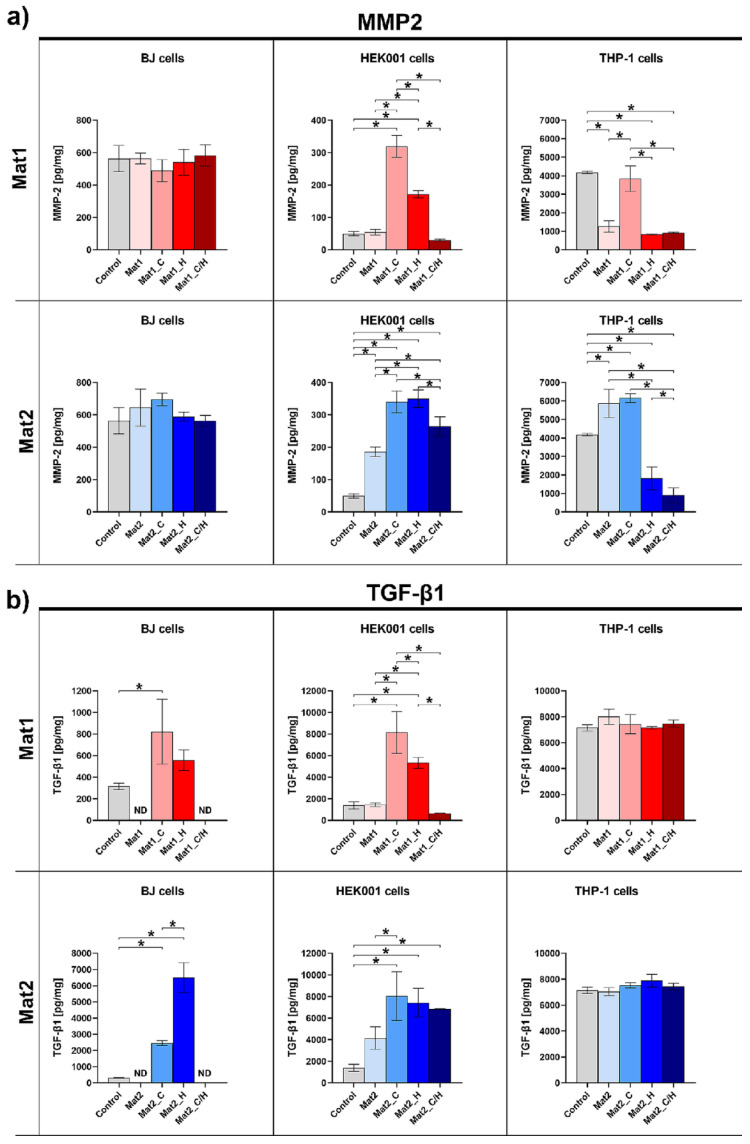
ELISA evaluation of (**a**) MMP-2 and (**b**) TGF-β1 levels in human skin fibroblasts (BJ cell line), epidermal keratinocytes (HEK001 cell line), and macrophages (derived from THP-1 cell line) cultured in the presence of biomaterials (control–cells culture in the inserts without biomaterial); * statistically significant results between indicated groups, *p* < 0.05, one-way ANOVA followed by Tukey’s test (Mat1: curdlan/agarose biomaterial, Mat2: curdlan/chitosan biomaterial, C–vitamin C, H–hydrocortisone, C/H–mixture of vitamin C and hydrocortisone, ND–not detected).

As shown in Figure 5a, all variants of biomaterials did not significantly impact MMP-2 synthesis by human skin fibroblasts. Nevertheless, Mat1 containing vitamin C (Mat1_C) and hydrocortisone (Mat1_H) significantly increased MMP-2 production by human keratinocytes compared to other samples. In the case of Mat2 material, all its variants significantly increased the MMP-2 level in keratinocyte culture compared to the control (cells cultured without biomaterial), but the greatest stimulatory effect was observed for the samples modified with bioactive agents. Importantly, all samples containing hydrocortisone (Mat1_H, Mat1_C/H, Mat2_H, and Mat2_C/H) significantly decreased release of MMP-2 by human macrophages. Hydrocortisone was proven to suppress pro-inflammatory transcription factor (nuclear factor-κB–NF-κB) and to increase inhibitor κB (IκB) in mononuclear cells. Moreover, Aljada et al. demonstrated that it reduces the level of MMP-2 in the plasma due to suppression of intranuclear activator-protein-1 (AP-1)–a modulator of MMP-2 genes [41]. Thus, observed decline in MMP-2 production by macrophages after exposure to the hydrocortisone-enriched biomaterials seems fully justified by previous reports available in the literature. Interestingly, vitamin-C-enriched biomaterials (Mat1_C, Mat2_C) and unmodified Mat2 that all had slightly acidic pH due to the presence of ascorbic acid and/or acetic acid used for the production–caused elevated MMP-2 release compared to the control (Mat2 and Mat2_C) or compared to hydrocortisone-enriched samples or unmodified Mat1 material (in the case of Mat1_C). It should be noted that slightly acidic extracellular pH (5.4–6.5) was demonstrated to induce MMP release in tumor tissues, which allows proteolytic degradation of ECM enabling tumor metastasis and angiogenesis [42]. Therefore, increased synthesis of MMP-2 by macrophages cultured in the presence of Mat1_C, Mat2_C, and Mat2 may be explained by their slightly acidic pH that had stimulatory effect on MMP expression. The pH impact may also explain high MMP-2 release by keratinocytes exposed to all variants of Mat2 (produced using acetic acid) and Mat1 modified with ascorbic acid (Mat1_C). Prolonged inflammation and elevated MMP production by immune cells are major factors responsible for chronicity of the wounds. Thus, both curdlan-based materials enriched with hydrocortisone and vitamin C/hydrocortisone, which significantly reduced MMP-2 synthesis by macrophages, appear to have potential in the treatment of chronic wounds, preventing pathological ECM degradation.

TGF-β1 is one of the most important pro-healing growth factors involved in maintaining skin homeostasis by participation in the regulation of many processes during wound regeneration; e.g., fibroblast and keratinocytes proliferation, collagen synthesis, angiogenesis [43,44]. Within these studies, human fibroblasts and keratinocytes exhibited significantly elevated level of TGF-β1 after exposure to Mat1 and Mat2 modified with vitamin C and hydrocortisone (Figure 5b). Interestingly, in general, immobilization of the mixture of vitamin C/hydrocortisone within biomaterials did not have positive impact on TGF-β1 release by skin cells. All variants of produced biomaterials did not reveal any impact on TGF-β1 release by macrophages. TGF-β1 participates in the re-epithelization and is crucial for successful wound closure. Chronic wounds show attenuated TGF-β1 signaling causing undesirable consequences, such as increased activity of inducible nitric oxide synthase (iNOS) and excessive NO synthesis [44]. Thus, application of wound dressings having the ability to increase TGF-β1 expression is very beneficial in the treatment of non-healing chronic and burn wounds.

## 3. Materials and Methods

### 3.1. Materials

Curdlan (from *Alcaligenes faecalis*; specific rotation [A]20/D: +30∼+35; DP 6790) was purchased from Wako Pure Chemicals Industries (Osaka, Japan). Agarose (low EEO, gel point 36 ± 1.5 °C), chitosan (75% deacetylation degree, 50–190 kDa molecular weight, viscosity 20–300 cP), 3-O-ethyl-L-ascorbic acid (vitamin C), hydrocortisone, phorbol myristate acetate (PMA), 2-mercaptoethanol, penicillium, streptomycin, MTT reagent 3-(4,5-dimethylthiazol-2-yl)-2,5-diphenyltetrazolium bromide), Live/Dead Double Staining Kit, DAPI, and Cell Counting kit-8 were provided by Sigma-Aldrich Chemicals (Warsaw, Poland). Acetic acid and sodium hydroxide were obtained from Avantor Performance Materials (Gliwice, Poland). Phosphate buffered saline (PBS) and fetal bovine serum (FBS) were obtained from Pan-Biotech GmbH (Aidenbach, Germany). ELISA kit specific to human type I collagen (Col I) and ELISA kit for determination of general cortisol were obtained from EIAab (Wuhan, China). ELISA kits specific to human MMP-2 and TGF-β were obtained from Biorbyt (Cambridge, UK). BCA Protein Assay Kit was purchased from Thermo Fisher Scientific (Waltham, MA, USA). Primary human specific anti-type I collagen (Col1a1/Col1a2) antibodies were obtained from Abnova (Taipei, Taiwan). Secondary antibodies conjugated to AlexaFluor647 were purchased from Abcam (Cambridge, UK). Cell lines (normal human skin fibroblasts–BJ, normal human epidermal keratinocytes transformed with HPV-16–HEK001, and human acute monocytic leukemia cells–THP-1), Eagle’s Minimum Essential Medium (EMEM), and Roswell Park Memorial Institute medium (RPMI) were obtained from American Type Culture Collection (ATCC-LGC Standards, Teddington, UK). Keratinocyte-Serum Free medium, L-glutamine, and human recombinant EGF were obtained from Gibco, Life Technologies (Grand Island, NY, USA).

### 3.2. Biomaterials Fabrication

Novel biomaterials were produced by combining two methods: sol-gel method and thermal gelation of the biopolymers. To prepare curdlan/agarose biomaterial (marked as Mat1), 2% (*w*/*v*) curdlan and 2% (*w*/*v*) agarose were suspended in deionized water. To prepare curdlan/chitosan biomaterial (marked as Mat2), 2% (*w*/*v*) curdlan and 1% (*w*/*v*) chitosan were suspended in 1% (*v*/*v*) acetic acid solution. The obtained suspensions were subjected to preheating (50 °C) and mixing on a magnetic stirrer and transferred into a round-shape flat molds. The samples were heated for 20 min in a water bath at 95 °C for 20 min followed by sample cooling. Then, both Mat1 and Mat2 samples were subjected to freezing (at −80 °C) and freeze-drying (LYO GT2-Basic, SRK Systemtechnik GmbH, Riedstadt, Germany). After lyophilization process, resultant samples (Mat1 and Mat2) were soaked in PBS solution containing vitamin C at a concentration of 200 µg/mL^−1^ g of sample was soaked with 12.5 mL of the solution (samples marked as Mat1_C and Mat2_C) or in PBS solution containing hydrocortisone at a concentration of 5 µg/mL^−1^ g of sample was soaked with 12.5 mL of the solution (samples marked as Mat1_H and Mat2_H) or in PBS solution containing a mixture of vitamin C/hydrocortisone (200 µg/mL/5 µg/mL)^−1^ g of sample was soaked with 12.5 mL of the solution (samples marked as Mat1_C/H and Mat2_C/H). To ensure better detection, samples for vitamin C release test were soaked with ascorbic acid solution at higher concentration (500 µg/mL). Then, the soaked samples were frozen at −80 °C and subjected to freeze-drying.

### 3.3. Vitamin C and Hydrocortisone Release Profile

The release profile of vitamin C and hydrocortisone from the samples was evaluated using a flow-through procedure in a closed-loop system (USP4 Sotax drug release apparatus, Donau Lab, Dietikon, Switzerland). The samples weighing 120 ± 2 mg were inserted into USP4 flow-through cells. The 50 mL of PBS was used as an elution medium, which perfused at a rate of 3 mL per min at 37 °C. At predetermined time points, 1.5 mL samples were collected for the evaluation of vitamin C and hydrocortisone concentration and replaced with an equal amount of fresh PBS. The amount of vitamin C in collected samples was evaluated by measurement of absorbance at 252 nm using a UV-spectrophotometer (Genesys 6 UV-Vis, Thermo Fisher Scientific, Waltham, MA, USA). Concentrations of vitamin C in collected samples were determined using the absorbance values obtained with the calibration curve that was prepared applying known concentrations of vitamin C. Hydrocortisone concentrations were evaluated using ELISA kit. The vitamin C and hydrocortisone release profiles from the samples were expressed as cumulative concentration at predetermined time intervals

### 3.4. Cell Culture Experiments

Normal human skin fibroblasts (BJ), human epidermal keratinocytes transformed with HPV-16 (HEK001), and human acute monocytic leukemia cells (THP-1) were used in the experiments. BJ cells were cultured using EMEM supplemented with a 10% FBS. HEK001 cells were maintained in Keratinocyte-Serum Free medium containing 5 ng/mL human recombinant EGF and 2 mM l-glutamine. THP-1 cells were cultured in RPMI medium supplemented with 0.05 mM 2-mercaptoethanol and 10% FBS. To induce monocytic differentiation into mature macrophages, PMA at a concentration of 200 nM was added to RPMI medium. All culture media were additionally supplemented with streptomycin (0.1 mg/mL) and penicillin (100 U/mL). BJ, HEK001 and THP-1 cells were cultured at 37 °C in a humidified atmosphere with 5% CO_2_.

#### 3.4.1. Cytotoxicity Assessment

The cytotoxicity assessment was conducted in accordance with the procedure described in ISO 10993-5 [45] using 24 h extracts of biomaterials prepared according to ISO 10993-12 [46] (maintaining the ratio 25 mg sample/1 mL medium; polystyrene extract was considered as a negative control of cytotoxicity). In brief, BJ cells were seeded in 100 µL of the medium into 96-well plates at a concentration of 2 × 10^5^ cells/mL. After the 24 h incubation, the culture medium was replaced with extracts of biomaterials and the cells were incubated in the presence of the extracts for further 24 h. Then, extracts of biomaterials were discarded, cell monolayer was gently rinsed twice with PBS, and 100 µL of fresh culture medium containing 1 mg/mL of MTT reagent were added to each well. To assess cell viability, MTT colorimetric test was performed, as it was described previously [47]. The obtained results were shown as a percentage of absorbance value gained with negative control of cytotoxicity.

#### 3.4.2. Proliferation Assessment

The ability of biomaterials to support the proliferation of BJ cells was assessed by indirect contact method. In brief, biomaterials weighting 40 ± 2 mg were put into the wells of 12-well plate and pre-soaked in a complete culture medium. Then, cell culture inserts having semipermeable membrane were placed above the biomaterials and 1.5 mL of complete culture medium were added to the wells of 12-well plate (outside the insert). BJ cells were seeded into the inserts in 500 µL of culture medium at a concentration of 2 × 10^4^ cells/mL and cultured for 5-days at 37 °C. Cells cultured in the inserts without the biomaterials placed on the well bottom served as a control. On the 3rd day, a 500 µL of the medium was replaced with a fresh portion. At pre-determined time intervals, a number of cells in the inserts was evaluated using Cell Counting kit-8 (WST-8 test) as it was described previously [48]. Before performing WST-8 test, cell culture inserts were transferred to the new wells, gently rinsed twice with PBS, and fresh culture medium was added to each well.

#### 3.4.3. Cell Migration Assessment

Impact of biomaterials on cell migration was evaluated by wound healing assay (known also as scratch assay). In brief, BJ and HEK001 cells were seeded in 1 mL of the medium into the wells of 24-well plates at a density of 2 × 10^5^ cells/mL. After 48 h of culture, when cells reached approx. 95–100% confluence, a scratch or denuded region was created with a sterile pipette tip. Then, 750 µL of 24 h extracts of biomaterials were added to BJ and HEK001 cells. The biomaterials extracts were prepared according to ISO 10993-12 standard [46] (maintaining the ratio 25 mg sample/1 mL medium; polystyrene extract was considered as a control). After 24 h exposure to the extracts, the cells were stained using May-Grunwald–Giemsa dye according to the procedure described earlier [49]. Stained cells were visualized using inverted optical microscope (Olympus CKX53, Warsaw, Poland), and then cell migration (wound/scratch closure) was evaluated according to the procedure described previously [50].

#### 3.4.4. Type I Collagen Production Assessment

BJ cells were cultured in the presence of biomaterials in a two-compartment environment using cell culture inserts as described in Section 3.4.2. After 5-day culture, the amount of type I collagen (Col I) was evaluated in cell lysates using ELISA kit specific to human Col I. The preparation of cell lysates was conducted according to the procedure described earlier by repeated freeze-thaw cycles, followed by ultrasonication [51]. The level of Col I was normalized to the total cellular proteins determined by BCA Protein Assay Kit and expressed as ng of collagen per mg of total cellular proteins. Additionally, Col I deposition was visualized by immunofluorescence staining according to the procedure described earlier [50] and observed under confocal laser scanning microscope (CLSM; Olympus Fluoview equipped with FV1000, Olympus, Tokyo, Japan).

#### 3.4.5. MMP-2 and TGF-β1 Production Assessment

BJ fibroblasts, HEK001 keratinocytes, and THP-1-derived macrophages were cultured in the presence of biomaterials in a two-compartment environment using cell culture inserts as described in Section 3.4.2. After 5-day culture, the cell culture supernatants were collected to evaluate concentrations of MMP-2 and TGF-β1 by ELISA kits specific to human MMP-2 and TGF-β1. The level of MMP-2 and TGF-β1 was normalized to the total cellular proteins.

### 3.5. Statistical Analysis

The obtained results were expressed as mean values ± standard deviation (*n* = 3). Statistical analysis was carried out using one-way analysis of variance (ANOVA) followed by Tukey’s multiple comparison test (GraphPadPrism 8.0.1 Software, Inc., San Diego, CA, USA). Statistical significance was considered at *p* values less than 0.05.

## 4. Conclusions

The studies revealed that developed bioactive curdlan-based biomaterials (curdlan/agarose–Mat1 and curdlan/chitosan–Mat2) enriched with vitamin C and/or hydrocortisone were characterized by burst vitamin C release profile and gradual release (for 6–8 days) of low doses of the hydrocortisone, which is especially beneficial during management of burn wounds with hypergranulation tissue and excessive ROS concentration at the wound bed. Among all tested variants of biomaterials, dressings enriched with hydrocortisone and the mixture of vitamin C/hydrocortisone showed the best therapeutic potential. Mat1_H and Mat1_C/H had the ability to significantly reduce MMP-2 synthesis by macrophages. Mat1_H additionally increased TGF-β1 release by skin cells, whereas Mat1_C/H positively affected fibroblast migration and proliferation. Different biological activities were observed for Mat2_H and Mat2_C/H biomaterials that not only significantly reduced MMP-2 synthesis and increased TGF-β1 production, but also stimulated type I collagen deposition. All mentioned biological activities of the biomaterials are much desired during management of chronic and burn wounds that are characterized by elevated level of MMPs, attenuated TGF-β1 signaling, and impaired healing. Thus, modification of previously developed curdlan-based biomaterials with bioactive compounds significantly improved their biological properties and clinical usefulness. Nevertheless, in-vivo experiments need to be performed to fully confirm their high biomedical potential.

## 5. Patents

The method for the production of Mat1 and Mat2 biomaterials is protected by Polish Patent no. 236367 and patent application no. P.430455, respectively.

## Data Availability

The raw/processed data required to reproduce these findings can be obtained from the corresponding author (agata.przekora@umlub.pl) upon reasonable request.
